# Profil des cancers gynécologiques et mammaires à Yaoundé - Cameroun

**DOI:** 10.11604/pamj.2014.17.28.3447

**Published:** 2014-01-17

**Authors:** Zacharie Sando, Jovanny Tsuala Fouogue, Florent Ymele Fouelifack, Jeanne Hortence Fouedjio, Emile Telesphore Mboudou, Jean Louis Oyono Essame

**Affiliations:** 1 Département des sciences morphologiques de la Faculté de Médecine et des Sciences Biomédicales de l'Université de Yaoundé 1, Cameroun; 2 Chef Service de l'unité d'anatomie pathologique de l'Hôpital Gynéco-Obstétrique et Pédiatrique de Yaoundé, Cameroun; 3 Département de Gynécologie et obstétriques de la Faculté de Médecine et des Sciences Biomédicales de l'Université de Yaoundé I, Cameroun; 4 Unité de Gynécologie et d'Obstétrique de l'Hôpital Central de Yaoundé, Cameroun; 5 Chef de l'Unité de Gynécologie et Obstétriques de l'Hôpital Gynéco-Obstétrique et Pédiatrique de Yaoundé-Cameroun; 6 Chef de département des sciences morphologiques de la Faculté de Médecine et des Sciences Biomédicales de l'Université de Yaoundé 1, Cameroun

**Keywords:** Cancer, sein, col, utérus, endomètre, ovaire, gynécologique, vagin, vulve, Cameroun, Cancer, breast, cervix, uterus, endometrium, ovary, gynecological, vagina, vulva, Cameroon

## Abstract

**Introduction:**

En Afrique subsaharienne, les cancers constituent un fléau dont les caractéristiques restent à préciser.

**Méthodes:**

Afin de déterminer les aspects histologiques et cliniques des cancers gynécologiques et mammaires au Cameroun, nous avons mené une étude descriptive et rétrospective sur une période de 54 mois à l'Hôpital Gynéco-Obstétrique et Pédiatrique de Yaoundé.

**Résultats:**

Les 424 cas enregistrés se répartissaient ainsi: cancers du col de l'utérus: 210 cas (49.5%); du sein: 144 cas (34%); de l'ovaire: 31 cas (7.4%); de l'endomètre: 21 cas (4.9%); de la vulve: 14 cas (3.3%); du vagin: 1 cas (0.2%) et les sarcomes utérins: 3 cas (0.7%). Pour le cancer du sein, l’âge moyen au diagnostic était de 46.08±4.0 ans, 92.4% de patientes présentaient une masse (dont 60.9% localisées au quadrant supéro-externe), 76.4% étaient découverts aux stades T3 et T4, et 71.5% étaient les carcinomes canalaires. Pour les cancers du col, l’âge moyen au diagnostic était de 52.43±3.82 ans, 62.9% étaient découverts aux stades FIGO 1 et 2, et 87.6% étaient des carcinomes épidermoïdes. Pour le cancer de l'ovaire, l’âge moyen au diagnostic était de 49.0±9.31 ans, 90.3% étaient des tumeurs épithéliales et 74.2% étaient aux stades 2 et 3 (FIGO). Quant aux cancers de l'endomètre, l’âge moyen au diagnostic était de 59±14.55 ans, 90.5% étaient des adénocarcinomes.

**Conclusion:**

Les principaux cancers étaient ceux du col de l'utérus et du sein. Le diagnostic étant souvent fait aux stades tardifs et par conséquent de mauvais pronostic, la prévention des cancers gynécologiques et mammaires devrait être renforcée au Cameroun.

## Introduction

Les pays en développement enregistrent 72% des décès dus au cancer dans le monde [1]. Ce fléau constitue la troisième cause de mortalité dans ces pays [2]. Les cancers gynécologiques représentent 19% des cancers dans le monde. En Afrique les cancers de la femme les plus fréquents sont ceux du sein et du col de l'utérus [3]. Au Cameroun, selon le Comité National de Lutte contre le Cancer, l'incidence annuelle des cancers diagnostiqués par l'histologie est passée de 1000 à 12000 cas entre 1992 et 2008. Quarante neuf pour cent de ces cancers ont été diagnostiqués chez les femmes [4] avec une nette prédominance des cancers gynécologiques [5]. Non seulement un seul cancer sur dix fait l'objet d'un diagnostic histologique, mais il n'existe pas de registre national du cancer. Ainsi les caractéristiques cliniques et histopathologiques des cancers sont peu décrites au Cameroun. L'objectif de ce travail était de déterminer les aspects histopathologiques et cliniques des cancers gynécologiques et mammaires de la femme à Yaoundé. Ceci permettrait de mieux orienter les politiques de prise en charge des cancers au Cameroun.

## Méthodes

L’étude était rétrospective et descriptive. Elle a été menée à l'Hôpital Gynéco-Obstétrique et Pédiatrique de Yaoundé (HGOPY) sur une période de quatre ans et six mois (du 1er juillet 2003 au 31 décembre 2008). Nous avons colligé les dossiers des femmes prises en charge pour un cancer du sein ou du tractus génital ayant fait l'objet d'un diagnostic anatomopathologique. L'HGOPY est un hôpital de référence spécialisé dans la prise en charge des cancers de la femme au Cameroun.

Les données cliniques contenues dans chaque dossier étaient collectées et reportées sur une fiche technique anonyme. Pour les cancers du sein nous avons relevé: l’âge, le côté, la parité, l'aspect cutané, le siège anatomique, les principaux symptômes, le stade « Tumour Nodes Metastasis » (TNM), le grade histopronostique de Scarff, Bloom et Richardson (SBR) et le type histologique. Pour les cancers génitaux nous avons relevé: l’âge, la parité, les signes cliniques, le stade clinique de Fédération Internationale des Gynécologues - Obstétriciens (FIGO), le stade « pathology Tumour » (pT) et le type histologique. Une clairance éthique avait été obtenue et les données analysées grâce aux logiciels « Statistical Package for Social sciences » (SPSS) version 10.1 et Microsoft Excel 2007.

## Résultats

Nous avons colligé 424 dossiers. Les données étaient reparties comme le montrent les différents tableaux et figures ci-dessous:

### Données sociodémographiques

**Antécédents familiaux des cancers du sein, de l'ovaire et de l'endomètre** Les antécédents familiaux de cancers ovariens, mammaires et endométriaux des patientes sont présentés dans le Tableau 1. Les patientes atteintes de cancer présentaient dans 12.64% (23 patientes sur 182) des cas un antécédent familial de cancer du même organe.

**Tableau 1:** Répartition des cancers du sein, de l'ovaire et de l'endomètre selon les antécédents familiaux de cancer

Organes	Antécédent Familial De Cancer		Total
	Oui	Non	
Sein	18 (9,9%)	115 (63,19%)	133 (73,07%)
Ovaire	3 (1,65%)	27 (14,84%)	30 (16,48%)
Endomètre	2 (1,1%)	17 (9,43%)	19 (10,45%)
Col de l'utérus	ND	ND	ND
Vulve	ND	ND	ND
Sarcome utérin	ND	ND	ND
Vagin	ND	ND	ND
Total	23 (12,64%)	159 (87,36%)	182 (100%)

ND = données non disponibles

**Figure 1:**
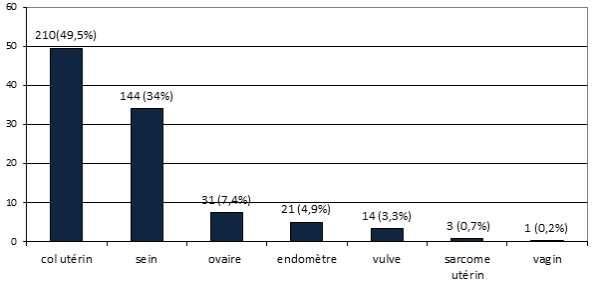
Distribution des patientes selon les organes atteints

**Distribution des patientes selon les organes atteints:** La Figure 1 représente la répartition des patientes selon les organes atteints de cancer. Les cancers du col de l'utérus et ceux du sein étaient les plus fréquents avec respectivement 49.5 (210 cas) et 34% (144 cas) des cancers.

**Distribution des patientes par tranches d’âge pour chaque organe atteint:** La répartition des patientes par tranches d’âge et par organe atteint est représentée dans le Tableau 2. Les cancers sont plus fréquents à partir de 40 ans et plus.

**Tableau 2:** Distribution des patientes par tranches d’âge et par organe atteint

Organes	Tranches d’âge						Total
	≤ 19 ans	20 - 29 ans	30 – 39 ans	40 - 49 ans	50 - 59 ans	≥ 60 ans	
Col	0	2 (0,47%)	22 (5,2%)	65 (15,37%)	60 (14,18%)	61(14,42%)	210 (40,64%)
Sein	1 (0,24%)	16 (3,78%)	29 (6,86%)	44 (10,4%)	28 (6,62%)	26 (6,15%)	144 (34,05%)
Ovaire	0	3 (0,7%)	6 (1,42%)	4 (0,95%)	11(2,6%)	7 (1,65%)	31 (7,32%)
Endomètre	0	0	0	3 (0,7%)	6 (1,42%)	12 (2,83%)	21 (4,96%)
Vulve	0	0	1 (0,24%)	4 (0,95%)	5 (1,18%)	4 (0,95%)	14 (3,3%)
Utérus (Sarcomes)	0	0	0	2 (0,47%)	1 (0,24%)	0	3 (0,71%)
Vagin	0	0	0	0	0	1 (0,24%)	1 (0,24%)
Total	1 (0,24%)	21 (4,95%)	58 (13,72%)	122 (28,84%)	111 (26,24%)	111 (26,24s%)	424 (100%)

**Parité des patientes selon l'organe atteint** Nous présentons dans le Tableau 3 la parité des patientes pour chaque organe atteint. La fréquence des cancers augmente avec la parité.

**Tableau 3:** Répartition des patientes selon la parité et l'organe atteint

Organes	Parité			Total
	0	1 – 4	5 et plus	
Col	2 (0,47%)	67 (15,8%)	141 (33,25%)	210 (49,53%)
Sein	7 (1,65%)	89 (20,99%)	48 (11,32%)	144 (33,96%)
Ovaire	3 (0,71%)	15 (3,54%)	13 (3,07%)	31 (7,31%)
Endomètre	1 (0,24%)	8 (1,89%)	12 (2,83%)	21 (4,95%)
Vulve	0	9 (2,12%)	5 (1,18%)	14 (3,30%)
Sarcome utérin	1 (0,24%)	2 (0,47%)	0	3 (0,71%)
Vagin	0	0	1 (0,24%)	1 (0,24%)
TOTAL	14 (3,30%)	189 (44,58%)	217 (51,18%)	424 (100%)

### Données cliniques

**Figure 2:**
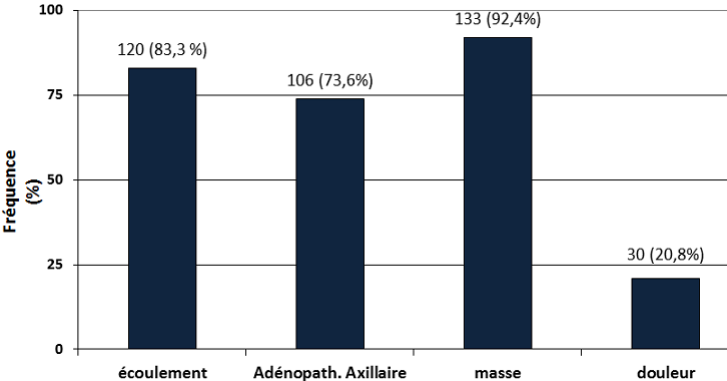
Les signes fonctionnels des cancers du sein

**Symptômes des cancers du sein:** La Figure 2 représente les symptômes présentés par les patientes atteintes de cancer du sein. Les signes les plus fréquents étaient la présence d'une masse du sein (92.4%), d'un écoulement (83.3%) et des adénopathies (73.6%).

**Coté du sein atteint**: Le sein droit était autant atteint que le sein gauche soit 64 cas (44.4%) chacun. L'atteinte était bilatérale dans 16 cas (11.1%).

**Figure 3:**
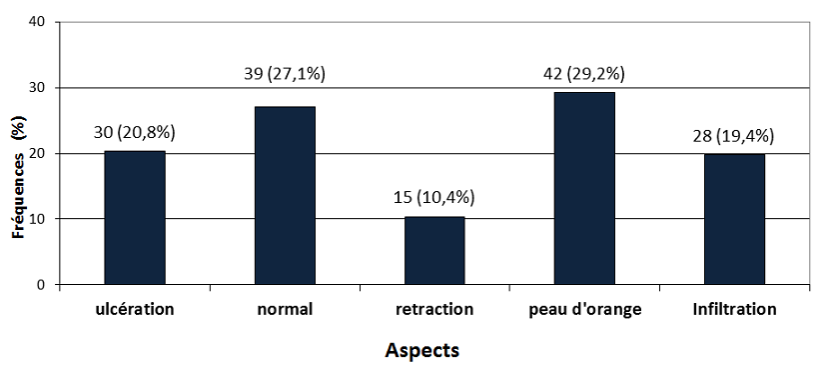
Répartition des cancers du sein selon l'aspect cutané

**Aspects cutanés des seins atteints:** Les aspects cutanés étaient repartis comme le montre la Figure 3. Le signe cutané prédominant était la peau d'orange présente dans 42 cas sur 144 (soit 29.2%).

**Figure 4:**
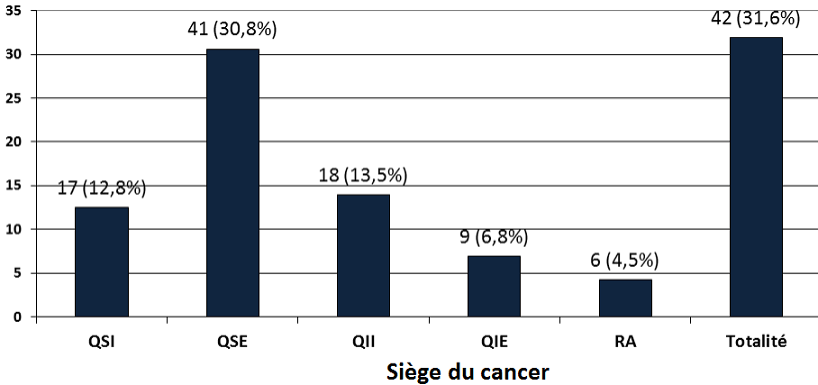
Répartition des patientes selon le siège anatomique du cancer du sein. (QII: Quadrant Inféro-Interne; QIE: Quadrant Inféro-Externe; QSE: Quadrant Supéro- Externe; QSI: Quadrant Supéro-interne; RA: retro-aréolaire)

**Topographie des masses cancéreuses sur les seins**: Les sièges topographiques des masses sur les seins étaient repartis comme le montre la Figure 4. Le quadrant supéro-externe était le plus atteint (41 cas sur 133 soit 31.8%), suivi des quandrants inféro-interne (13.5%) et supéro interne (12.8%).

**Figure 5:**
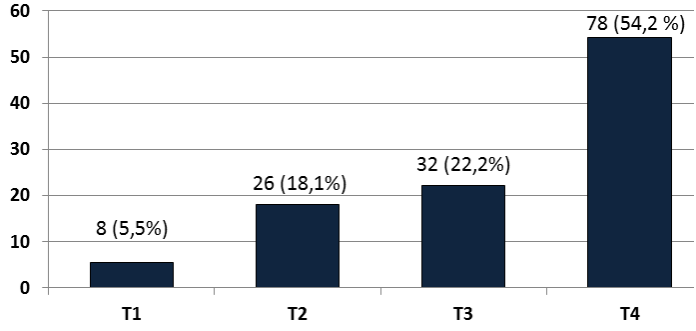
Répartition des patientes selon le stade “T” clinique des cancers du sein

**Le stade T (tumour) clinique des cancers du sein:** Les stades T des cancers du sein se représentés dans la Figure 5. Le stade T4 représentait 54.2% (78 cancers sur 144) tandis que le stade T1 ne représentait que 5.5% (8 cancers sur 144) des cancers.

**Stadification TNM des cancers du sein:** La répartition des cancers du sein selon le stade clinique TNM montrait que: des adénopathies étaient palpables dans 73.6% (106 cas sur 144) des cancers du sein dont 54.2% (78 cas) étaient au stade T4. Seuls 6.25% (9 cas sur 144) des cancers du sein présentaient des métastases à distance cliniquement évidentes au moment du diagnostic.

**Figure 6:**
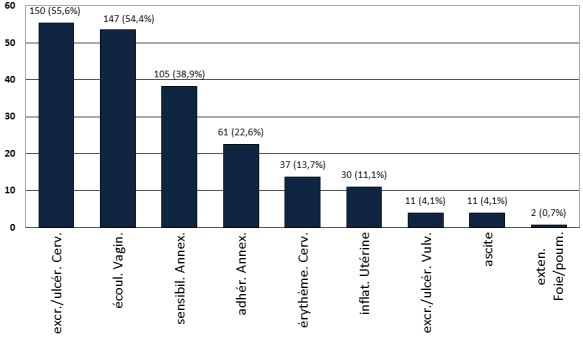
Les signes cliniques des cancers génitaux. Excr./ulcér. (Cerv.: excroissance ou ulcération du col de l'utérus – écoul. Vagin.: écoulement vaginal – sensibil. Annex.: sensibilité des annexes utérines – adhér. annex.: empâtement annexiel – érythème cerv.: érythème cervical – inflat. utérine: augmentation de taille de l'utérus - Excr./ulcér. vulv.: excroissance ou ulcération de la vulve – exten. foie/poumons: extension au foie ou aux poumons)

**Symptômes et signes cliniques des cancers génitaux:** Les manifestations cliniques des cancers génitaux sont résumées dans la Figure 6. Les excroissances du col et les écoulements vaginaux étaient les signes les plus fréquents.

**Stades FIGO des cancers génitaux:** Le Tableau 4 présente les stades FIGO des cancers génitaux dans notre série. Le Stade II de la FIGO était le plus représenté avec 37.04% (100 sur 270) des cas

**Tableau 4:** Distribution selon le stade FIGO des cancers génitaux

Organes	Stade FIGO												Total
	0	I-A	I-B	I-C	II-A	II-B	II-C	III-A	III-B	III-C	IV-A	IV-B	
**Col**	7 (2,59%)	8 (2,96%)	49 (29,26%)	4 (1,48%)	25 (9,26%)	43 (15,93%)	3 (1,11%)	26 (9,63%)	29 (10,74%)	0	10 (3,70%)	0	210 (77,78%)
**Ovaire**	0	1 (0,37%)	0	2 (0,74%)	8 (2,96%)	4 (1,48%)	0	10 (3,70%)	0	2 (0,74%)	3 (1,11%)	1 (0,37%)	31 (11,48%)
**Vagin**	0	0	0	0	1 (0,37%)	0	0	0	0	0	0	0	1 (0,37%)
**Vulve**	0	1(0,37%)	0	1 (0,37%)	7 (2,59%)	0	1 (0,37%)	4 (1,48%)	0	0	0	0	14 (5,83%)
**Endomètre**	0	6 (2,22%)	2 (0,74%)	2 (0,74%)	8 (2,96%)	0	0	1 (0,37%)	0	0	1 (0,37%)	1 (0,37%)	21(7,78%)
**sarcome utérin**	0	0	0	0	0	0	0	3 (1,11%)	0	0	0	0	3(1,11%)
**TOTAL**	**7(2,59%)**	**16 (5,92%)**	**51(18,89%)**	**9(3,33%)**	**49(18,15%)**	**47 (17,41%)**	**4(1,48%)**	**43 (15,93%)**	**29(10,74%)**	**2 (0,74%)**	**14 (5,19%)**	**2 (0,74%)**	**270 (100%)**

### Données histopathologiques

**Figure 7:**
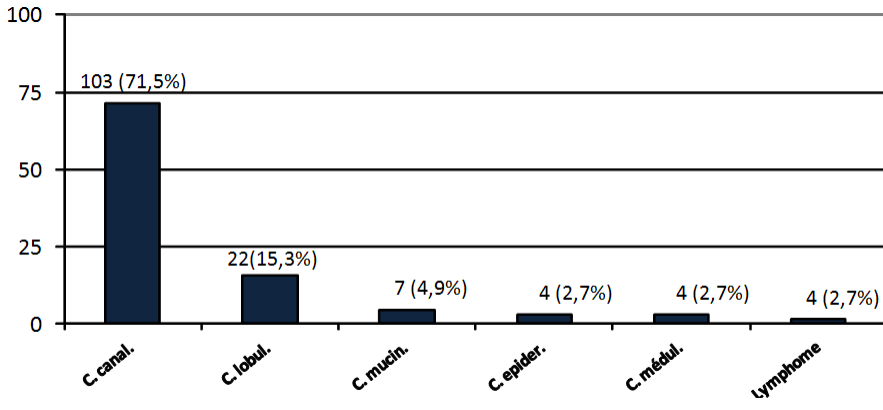
Distribution des patientes selon les types histologiques des cancers du sein (C. canal.: Carcinone canalaire – C. lobul.: Carcinome lobulaire - C. epider.: carcinome épidermoïde – C. mucin.: carcinome mucineux - C. médul.: carcinome médullaire – Lymph.: Lymphome)

**Types histologiques des cancers du sein:** Les types histologiques des cancers du sein étaient repartis comme le montre la Figure 7. Les carcinomes canalaire et lobulaire étaient les plus fréquents avec respectivement 71.4% (103 cas sur 144) et 15.3% (22 cas sur 144) de cas.

**Grade SBR des cancers du sein:** Le grade 2 de Scarff, Bloom et Richardson représentait 69,4% (100 cas sur 144) des cas; les grades 1 et 3 constituaient respectivement 23.6% (34 cas) et 7% (10 cas) des cancers du sein de notre série.

**Stade pT des cancers génitaux:** Le Tableau 5 résume les stades pT des cancers génitaux.

**Tableau 5:** Répartition des patientes selon le stade pathologique (pT)

Organes	Stade Pathologique (Pt)					Total
	Non Disponible	pT1	pT2	pT3	pT4	
**Col**	50(11,79%)	82(19,33%)	27 (6,37%)	28(6,60%)	23 (5,42%)	210(49,53%)
Sein	34 (8,02%)	15 (3,54%)	30 (7,08%)	20 (4,72%)	45(10,61%)	144(33,96%)
Ovaire	4 (0,94%)	4 (0,94%)	13 (3,07%)	8 (1,89%)	2 (0,47%)	31 (7,31%)
Endomètre	7 (1,65%)	6 (1,42%)	1 (0,24%)	5 (1,18%)	2 (0,47%)	21 (4,95%)
Vulve	7 (1,65%)	1 (0,24%)	6 (1,42%)	0	0	14(3,30%)
Sarcome utérin	0	2 (0,47%)	1(0,24%)	0	0	3 (0,71%)
Vagin	1(0,24%)	0	0	0	0	1 (0,24%)
**Total**	**103 (24,29%)**	**110 (25,94%)**	**78 (18,40%)**	**61 (14,39%)**	**72 (16,98%)**	**424 (100%)**

**Autres cancers:** Le seul cancer du vagin de notre série était un carcinome épidermoïde. Sur un total de 21 cancers de l'endomètre, 19 soit 90.5% étaient des adénocarcinomes et les 3 autres (9.5%) des carcinomes adéno-squameux. Nous avons enregistré 2 léiomyosarcomes et un sarcome stromal de l'utérus. Tous les 14 cancers de la vulve étaient des carcinomes épidermoïdes

**Types histologiques des cancers du col de l'utérus**: Les deux types histologiques des cancers du sein étaient le carcinome épidermoïde avec 87.6% (184 des 210 cas) des cas et l'adénocarcinome avec 12.4% (soit 26 des 210 cas) des cas.

**Types histologiques des cancers de l'ovaire**: Les types histologiques des tumeurs ovariennes sont présentés dans le Tableau 6. Les tumeurs épithéliales constituaient 90.32% (soit 29 des 31 cas) des cancers de l'ovaire de notre série.

**Tableau 6:** Répartition selon le type histologique des cancers de l'ovaire

Types histologiques	Fréquences
**Tumeurs épithéliales**	28 (90,32%)
Cystadénocarcinome papillaire séreux	18 (58,06%)
Cystadénocarcinome mucineux	7 (22,58%)
Adénocarcinome à cellules claires	3 (9,68%)
**Tumeurs du mésenchyme et des cordons sexuels**	**2 (6,46%)**
Tumeur à cellules de la granulosa	1 (3,23%)
Tumeur de Sertoli-Leydig	1 (3,23%)
**Tumeurs germinales**	1 (3,23%)
Tératome immature	1 (3,23%)
**Total**	**31 (100%)**

## Discussion

### Aspects épidémiologiques/sociodémographiques et cliniques

**Le cancer du col:** En zone urbaine au Cameroun, le cancer du col représentait 11 à 31.74% [4, 6–8] des cancers de la femme il y a deux décennies et 40.18% [9] des cancers gynécologiques et mammaires il y a 10 ans. Des travaux préliminaires récents réalisés en zone semi-urbaine ont retrouvé 57% de cancers du col parmi les cancers gynécologiques [10]. Les données disponibles sur les aspects cliniques et histologiques des cancers du col de l'utérus sont limitées aux formations hospitalières. Notre étude a porté sur les cancers du col pris en charge à l'Hôpital national de référence en gynécologie oncologique ce qui lui confère à peu près un caractère représentatif de l'ensemble du pays. Le cancer du col était le plus fréquent avec une proportion de 49.5% (210 cas sur 424) des cancers gynécologiques et mammaires (Figure 1). Cette proportion était de 43.65% en Afrique subsaharienne [6]. La nette prédominance du carcinome épidermoïde (184 cas sur 210 soit 87.6%) observée est également en accord avec les statistiques internationales. L’âge moyen était de 52.43±3.82 ans pour les cancers du col (Tableau 2), chiffre superposable aux âges moyens de 46 à 53 ans rapportés par de précédentes séries locales et étrangères tant dans les pays riches que dans les pays pauvres [6, 8, 9, 11–14]. Dans notre série 67.14% (141 des 210 cas) des cas ont été diagnostiqués chez des grandes multipares (Tableau 3). La multiparité est un facteur de risque connu du cancer du col de l'utérus [14]. Nous avons observé 62.9% (132 des 210 cas) de diagnostics tardifs aux stades FIGO 1 et 2 (Tableau 4), ceci s'explique par le long délai entre l'apparition des symptômes et le recours aux soins et l'absence de politique nationale de dépistage et de vaccination. En effet des enquêtes ont montré que les connaissances, attitudes et pratiques de femmes et du personnel de santé camerounais vis-à-vis du virus *HPV (Human Papilloma Virus)* et du cancer du col de l'utérus sont faibles [15–17]. Le dépistage par l'inspection visuelle après application de l'acide acétique est limité à quelques hôpitaux de références, pourtant c'est désormais la méthode de choix dans les pays pauvres [18, 19]. En raison de son coût prohibitif et le taux de sensibilisation faible, la vaccination anti-HPV n'est pas encore largement utilisée au Cameroun.

**Le cancer du sein:** Le cancer du sein était le second de notre série avec une proportion de 34% (Figure 1) derrière le cancer du col de l'utérus (49.5%) comme c'est le cas en Afrique subsaharienne où ces 2 cancers représentent 45.4% des nouveaux cas de cancers de la femme chaque année [6]. Le cancer du sein est le plus prévalent des cancers de la femme dans le monde tant dans les pays développés que dans les pays en développement. Il représente 23% des cancers de la femme et 10,9% de tous les cancers humains au monde [6]. La maternité est l'un des facteurs protecteurs du cancer du sein [20] mais nous avons observé qu'elle était présente dans 95,8% (138 des 144 cas) des cas de notre série et que 33.33% (48 des 144 cas) des femmes atteintes de cancers du sein étaient des grandes multipares (Tableau 3); en effet le rôle protecteur de la grossesse ne concerne que les cancers survenant après la ménopause et de plus 50% des cancers du sein surviennent chez des femmes ne présentant aucun des facteurs de risque connu [20, 21]. S'agissant de la présentation clinique, la différence majeure concerne le stade au moment du diagnostic avec des cancers aux stades précoces dans les pays riches et des tumeurs avancées dans les pays à faibles revenus. Dans notre étude, 76.4% (110 de 144 cas) des cancers de ont été reconnus aux stades T3 et T4 (Figure 5) et 73.6% (106 des 144 cas) présentaient des adénopathies axillaires palpables (Figure 2). Ce retard au diagnostic est la conséquence des carences d'un système de santé très limité en ressources financière, matérielle et humaine et du bas niveau de connaissance des femmes vis-à-vis du cancer du cancer du sein. L'implémentation d'un programme de dépistage améliorerait le pronostic des patientes du cancer du sein. L’âge moyen au diagnostic était de 46,08±4,0 ans (Tableau 2), valeur similaire à celle rapportée en milieu rural [10]. Cent trente-trois cancers sur 144 (92.4%) présentaient sous la forme d'une masse localisée au quadrant supéro-externe du sein dans 60.9% (81 des 133 cas) de ces cas (Figure 4); des modifications cutanées étaient présentes dans 72.9% (105 cas sur 144) de nos cancers (Figure 3) dont 83.3% (120 des 144 cas) présentaient un écoulement (Figure 2). Les formes bilatérales représentaient 11.1% (16 cas sur 133) de l'effectif et 13.53% (18 des 133 cas) des femmes avaient un antécédent familial de cancer du sein (Tableau 1). Il a été établi que la prédisposition familiale s'explique par la mutation des gènes *BRCA 1 et 2 (Breast Receptor Cancer Antigen)* et qu'elle est responsable de 5 à 10% des cancers du sein [20–22].

**Le cancer de l'ovaire:** En Afrique sub-saharienne, le cancer de l'ovaire est le sixième cancer chez la femme et le troisième cancer de l'appareil reproducteur derrière ceux du sein et du col de l'utérus en Afrique sub-saharienne [2, 6]. Dans notre série le cancer de l'ovaire était aussi le troisième des cancers génitaux avec une proportion de 11.5%, soit 31 cas sur 270 (Figure 1). La multiparité est décrite comme un facteur protecteur du cancer de l'ovaire dont 10 à 12% ont une prédisposition génétique; nous avons observé que 3% de nos 31 patientes (9.7%) avaient un antécédent familial de cancer ovarien (Tableau 1). Quoique 41.94% (13 des 31 cas) des cas aient été observés chez des grandes multipares (Tableau 3) nous ne pouvons récuser le rôle protecteur de la multiparité à cause de la faible taille de notre échantillon. L’âge moyen au diagnostic dans notre série était de 49.0± 9.31 ans (Tableau 2) et conformément aux données de la littérature le diagnostic était tardif avec 16 cas sur 31 (51.61%) aux stades FIGO 3 et IV (Tableau 4, Tableau 5) [9, 10, 24, 25]. L'incidence maximale du diagnostic du cancer de l'ovaire dans les pays développés s'observe entre 65 et 74 ans; la différence avec notre série pourrait s'expliquer par l'espérance de vie de la femme camerounaise limitée à 57.1 ans [25].

**Le cancer de l'endomètre:** Le cancer de l'endomètre arrivait en quatrième position avec une proportion 4.9% (21 cas sur 424) loin derrière les cancers du sein et du col de l'utérus (Figure 1). Ceci est proche des chiffres rapportés dans les pays en développement [8–13]. Dans les pays développés, le cancer de l'endomètre est le cancer le plus fréquent des cancers génitaux féminins et son pic d'incidence se situe dans la septième décade; Dans notre étude 85.73% (soit 18 des 21 cas) des cas étaient diagnostiqués après 50 ans (Tableau 2) comme dans d'autres pays d'Afrique noire [26, 27].

**Les cancers de la vulve et du vagin:** Le cancer de la vulve comptait pour 3.3% (14 cas sur 421) et celui du vagin pour 0.2% (1 des 424 cas) des cas de notre série (Figure 1). Ces deux cancers sont ubiquitaires avec peu de différence d'incidence entre les régions, le cancer de la vulve représentant 5% et celui du vagin 3% des cancers gynécologiques. Leurs pics d'incidences se situent entre 60 et 70 ans [28], ce qui a été vérifié dans notre série avec une moyenne d’âge de 53.57 ± 9.15 ans pour le cancer de la vulve et 69 ans pour le cancer du vagin (Tableau 2)

### Aspects histologiques

**Le cancer du col:** Dans notre série 87.6% (184 des 210 cas) des cancers du col étaient des carcinomes épidermoïdes ce qui est conforme à la littérature. Le reste était constitué d'adénocarcinome (12.4%). Nous n'avons pas observé d'autres types histologiques faisant évoquer des localisations cervicales secondaires d'autres cancers [5–10].

**Le cancer de l'endomètre:** L'adénocarcinome était le type histologique le plus fréquent, représentant 19 des 21 cas (90.5%). Ceci corroborant les données de la littérature [30].

**Le cancer du sein** Conformément aux données de la littérature le carcinome canalaire était de loin le plus fréquent représentant 71.5% (103 cas sur 144) des cas (Figure 7). Le pronostic histologique selon Scarff, Bloom et Richardson était favorable (grade I) dans 23% (35 des 144 cas) des cas et intermédiaire (grade II) dans 69.4% (100 des 144 cas) des cancers de notre série; de façon globale donc les cancers du sein de notre série auraient eu un assez bon pronostic s'ils avaient été vus à des stades cliniques précoces, la recherche des récepteurs hormonaux et d'emboles tumoraux lymphatiques n’étant pas réalisée dans notre contexte [30].

**Le cancer de l'ovaire:** Les tumeurs épithéliales comptaient pour 90.32% (28 sur 31) des cas de notre série (Tableau 6), ce qui est classique s'agissant de l'histopathologie du cancer de l'ovaire [19, 26]. L'organisation mondiale de la santé a défini des critères préalables à l'organisation du dépistage systématique des maladies. Le cancer de l'ovaire ne remplissant pas lesdits critères, le diagnostic précoce passe un suivi gynécologique routinier.

**Les cancers de la vulve et du vagin:** S'agissant de l'histologie, tous les cancers de la vulve et du vagin que nous avons observés étaient des carcinomes épidermoïdes ce qui est similaire aux résultats d'autres série [28, 31]. La vulve étant un organe de surface elle est facilement accessibles aux investigations cliniques et paracliniques d'où les diagnostics précoces (stades FIGO 1 et 2) observés dans la 71.4% (10 des 14 cas) des cas de notre étude (Tableau 4).

## Conclusion

Les cancers du col de l'utérus et du sein étaient les plus fréquents (avec respectivement 49.5 et 34%). Les cancers des autres organes génitaux constituaient 16.3% des cas. Les types histologiques dominant étaient le carcinome épidermoïde pour le col de l'utérus et le carcinome canalaire pour le sein. Le diagnostic des cancers gynécologiques et mammaires reste tardif au Cameroun, aggravant ainsi le pronostic. Leur prévention devrait être renforcée
